# Active public involvement in healthcare associated infection (HCAI) research: developing collaborative projects

**DOI:** 10.1186/1753-6561-5-S6-P267

**Published:** 2011-06-29

**Authors:** A Whitfield, A Tingle, H Loveday

**Affiliations:** 1 National HCAI Research Network, Thames Valley University, London, UK

## Introduction / objectives

Partnership between service users and researchers is a cornerstone of the current research and development strategy for England’s National Health Service and is considered by funders as essential throughout the entire research process. To promote and facilitate public involvement in the field of HCAI research, the HCAI Research Network (RN) formed a Service User Research Forum (SURF) to inform priorities and contribute to HCAI research. This paper shows how SURF is supported to develop its own research priorities into viable projects.

## Methods

The HCAI RN has developed a robust process with SURF to identify priorities and develop feasible research questions, facilitate interrogation to assess viability and ethics and ensure potential benefits are defined. This enables lay researchers to contribute to literature reviews and desk research; determine appropriate funding; work in partnership with academic/ clinical researchers and undertake research team roles. The HCAI RN provides training and support for this process.

## Results

In 2010, SURF worked collaboratively with clinical and academic researchers to submit a proposal exploring patient experiences of MRSA screening for funding. The group is currently developing a user-led project evaluating health professional education in HCAI.

## Conclusion

Active service user involvement contributes diverse perspectives, ensures projects are relevant to patients and the public and empowers service users to make real contributions to the reduction of HCAI and drive change within healthcare settings. We continue to develop strategies to enable SURF involvement at all stages of research. Dedicated training and support ensures service users have the research skills and confidence to make their knowledge, experience and insights count in HCAI research.

## Disclosure of interest

None declared.

